# Dynamic behavior of a nematic liquid crystal mixed with CoFe_2_O_4_ ferromagnetic nanoparticles in a magnetic field

**DOI:** 10.3762/bjnano.8.246

**Published:** 2017-11-22

**Authors:** Emil Petrescu, Cristina Cirtoaje, Cristina Stan

**Affiliations:** 1 University Politehnica of Bucharest, Department of Physics, Splaiul Independenţei 313, 060042, Bucharest, Romania

**Keywords:** ferromagnetic nanoparticles, Fréedericksz transition

## Abstract

The dynamic behavior of a mixture of 4-cyano-4′-pentylbiphenyl (5CB) with 1% CoFe_2_O_4_ nanoparticles was analyzed. Experimental data indicate a high stability of the nematic director in the mixture compared to a reference 5CB sample in the magnetic field. The ferrite nanoparticles agglomerate forming long chains as observed in polarized microscopy images. These chains have a very high influence on the magneto-optic effect of the cell. When the magnetic field is applied on the mixture, the chains tend to align with the field direction but, due to their large size, they remain oriented obliquely between the support plates. Thus, the nematic molecules anchored on their surface can not reorient with the field and only a small distortion angle of the liquid crystal molecular director is observed. A comparison with a previously developed theoretical model confirms this small deviation.

## Introduction

Currently, there is a high interest in the theoretical analysis of suspensions of ferromagnetic particles in water, the so-called ferrofluids [1], or in liquid crystals [2–8], as well as in related experimental studies [9–10].

Optical phase modulation can be easily achieved in nematic liquid crystals (LC) subjected to external fields due to their crystalline order and liquid molecular mobility. Usually, electric fields are used for LC-based phase modulators because of their short response time and low voltages, which makes them applicable for compact devices. However, electric fields have some disadvantages related to secondary effects such as electrical polarisation and backflow. Because magnetic fields do not lead to polarization and exhibit an increased stability (low backflow) they can be a suitable alternative for these devices. There are two major reason why magnetic fields are not used in phase modulation. The first reason is the long response time of liquid crystals to the field action. The second reason is the high threshold for the Fréedericksz transition, which requires powerful DC voltage sources and large electromagnets.

By using ferromagnetic particles, the response time can be reduced, but at the cost of a higher Fréedericksz threshold [11–12]. The unique properties of nanoparticles open new development directions not only in materials science, but also in electronics, providing considerable improvement [13]. Liquid crystals, largely used for displays, are now mixed with nanoparticles such as magnetite, carbon nanotubes and quantum dots [14–22], yielding modified optical properties [23–25]. The main advantages of nanoparticles are given by their small dimensions and unique physical properties that strongly affect the local nematic order [26]. This leads to significant variations of transition thresholds [27–28] or response time [29]. Ferromagnetic nanoparticles are good candidates for LC improvement due to their significant positive magnetic anisotropy and strong anchoring energy [30–31] on a large surface provided by chain clustering. All these effects help the magnetic field to reorient the LC molecules and faster decrease the transition threshold.

A mixture of 1% CoFe_2_O_4_ and the nematic 4-cyano-4′-pentylbiphenyl (5CB, Aldrich) was used in a 180 micrometer thick planar cell subjected to an external magnetic field. Due to their magnetic properties, the nanoparticles are agglomerating in long chains on which LC molecules are attached like leaves on a tree branch. Previous results [32] and microscopy images indicate that CoFe_2_O_4_ nanoparticles are gathering in long chains which align themselves parallel to the rubbing directions indicating a strong anchoring to the support area. This new structure obtained inside the cell exhibits a different Fréedericksz transition and a shorter response time compared to a pure 5CB nematic cell.

## Theoretical background

**Figure 1:**
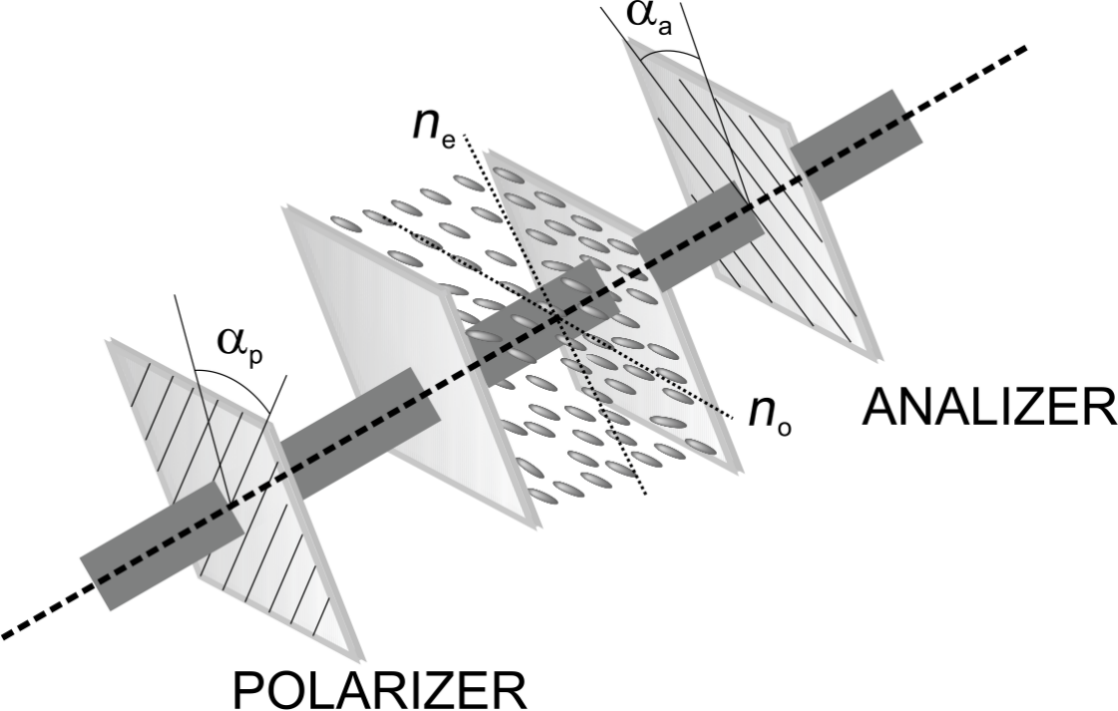
Schematic representation of a laser beam (λ = 632.8 nm) passing through a planar nematic cell placed between crossed polarizers.

Nematic liquid crystals are uniaxial crystals. When placed between crossed polarizers and passed through by a laser beam (Figure 1) they produce an intensity variation:

[1]
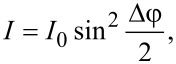


where Δφ = 2π*l*/λ is the phase difference between ordinary and extraordinary ray. Figure 1 shows a schematic representation of this system. The polarizer axis is at a 45° angle α_p_ relative to the polarization axis of incident laser beam, and the analyzer axis is perpendicular to the polarizer axis (α_p_ − α*_a_* = π/2).

The optical path difference (*l*) induced in a cell with the thickness *d* can be written as:

[2]
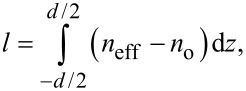


where *n*_o_ is the ordinary refractive index and *n*_eff_ is the effective refractive index. When the liquid crystal molecular director changes its orientation with an angle θ, we get (with the extraordinary refractive index *n*_e_):

[3]
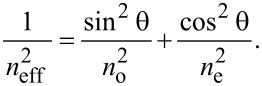


When 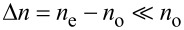 we get:

[4]
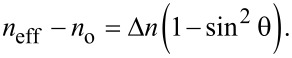


From Equation 2 and Equation 4 we obtain, using a small angles approximation:

[5]
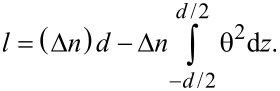


**Figure 2:**
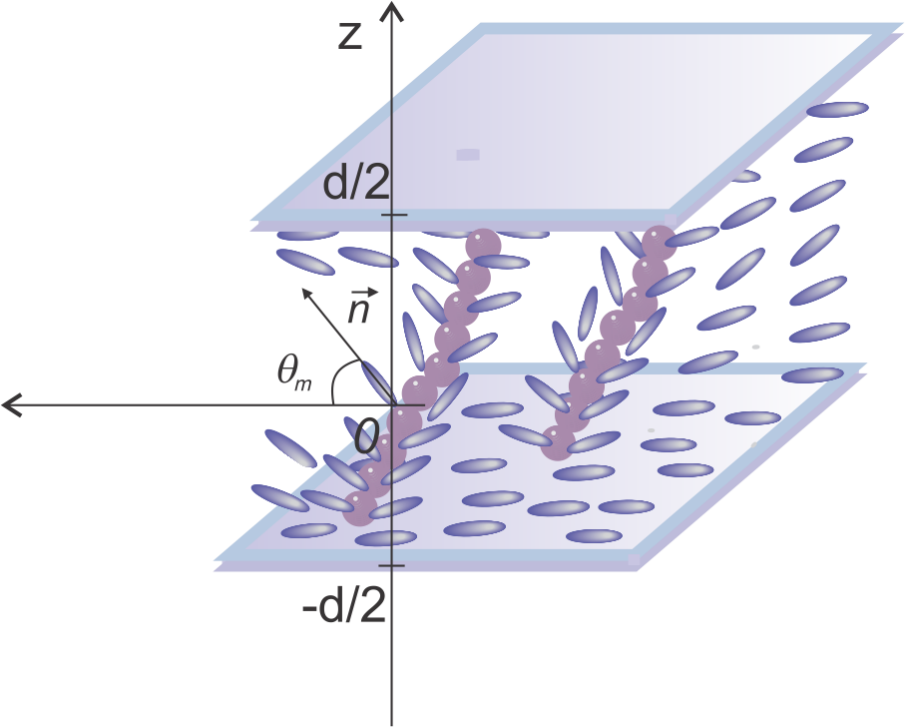
The deviation angle of molecular director inside a planar cell.

When the magnetic field is applied, the LC molecules change their orientation because of several factors: the interaction of the liquid crystal with the applied field, the interaction of the ferromagnetic nanoparticles with the magnetic field and the LC molecules anchoring on the surface of the added particles. The deviation angle θ between the glass plates is given by:

[6]
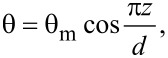


where *z* is the Oz coordinate of the LC molecules and θ_m_ is the maximum deviation angle (Figure 2).

On the glass support surface (*z* = *−d*/2 and *z* = *d*/2), the deviation angle has a minimum due to the anchoring forces on this surface. In the middle of the cell (*z* = 0) the deviation angle is maximum, because the molecules are out of the action range of the anchoring forces on the glass support are influenced by the ferroparticles and the magnetic field only. The maximum deviation angle θ_m_ is smaller than those obtained for pure LC, because of the movement limitation induced by the addition of the ferroparticles. Thus, we can still use the small angle approximation and obtain:

[7]
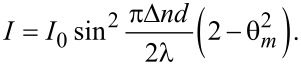


A theoretical characterization of distortion angle as a function of the time was made considering the model of a ferronematic liquid crystal in a magnetic field similar to the one proposed in [33]. By applying the elastic continuum theory, the free energy density of the systems is:

[8]
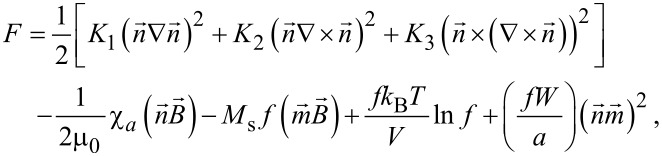


where *K*_1_, *K*_2_ and *K*_3_ are twist, splay and bend elastic constants, *M*_s_ is saturation magnetization, *V* is the particle volume, *a* is the average particle diameter, *m* is the magnetic moment of the particles, *W* is the surface density of the interfacial energy on the nematic boundary of the particles and *f* is the volume fraction of the magnetic particles.

Considering the dynamic evolution of a liquid crystal system as presented in [34], we can evaluate the temporal evolution of the distortion angle using the model similar to the one used in [30]. In this model, the term used for an azo-dye particle 
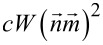
 is replaced with 
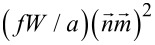
, the interaction term of the LC molecules with the surface of the ferroparticle. So, by applying the Euler–Lagrange equations and solving them in small angle approximation as presented in [9] we obtain the maximum deviation angle when the field is switched on:

[9]
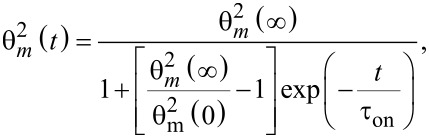


where 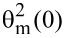 and 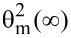 are the maximum deviation angles for *t* = 0 and *t* = ∞.

The time dependency of the deviation angle when the magnetic field is switched off is given by:

[10]
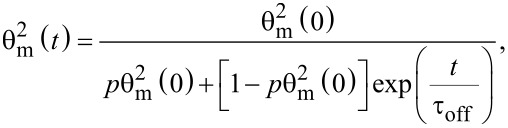


where *p* is a parameter depending on the elastic properties of the liquid crystal, the anchoring energy on the surface of the ferroparticle and the critical Fréedericksz threshold. Here τ_on_ and τ_off_ are the relaxation times for each case and they contain all the influences of the added ferroparticle and the applied external field as follows:

[11]
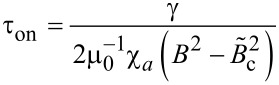


and

[12]
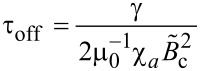


with

[13]
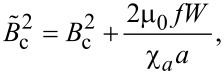


where *B* is the applied magnetic field, *B*_c_ is the Fréedericksz transition threshold for the pure liquid crystal and γ is the rotational viscosity of the liquid crystal. Thus, from Equation 9 and Equation 10, we obtain the time dependence of the emergent beam intensity and the influence of the added ferromagnetic nanoparticles on the response of the cell to the applied magnetic field.

## Results and Discussion

Dynamic measurements were performed with a reference sample cell filled only with 5CB and a cell filled with a mixture of 5CB and 1% (volumetric ratio) of CoFe_2_O_4_ nanoparticles. These nanoparticles are nano-sized ferrite platelets (30 nm diameter) obtained using a co-precipitation method with iron chloride and cobalt sulfate at a molar ratio of 2:1 in alkaline reaction medium (2 M NaOH) as described in detail in [20]. After the precipitated particles were separated from the reaction medium, they were repeatedly washed with deionized water. An aqueous solution of perchloric acid (25%) was added to coat particle surfaces in order to ensure an uniform colloidal suspension. The water was removed by evaporation and the nanoparticle powder was mixed with 5CB by repeated sonication until a homogeneous mixture was obtained. SEM analysis performed after water evaporation revealed quasi-uniform particles with spherical shape that tend to form associations when deposited on the sample support. The average diameter is approximately 40 nm. Magnetic properties were obtained from the magnetization curve in [35], where the saturation magnetization of CoFe_2_O_4_ was *M*_s_ = 58 Am^2^/kg corresponding to a magnetic field intensity of 790 kA/m and a coercive field of 239 kA/m. The cell thickness was set by a pair of 180 micrometer Mylar sheets and planar alignment was obtained by coating the glass plates with a solution of 0.1% PVA and rubbing them with a soft cloth.

**Figure 3:**
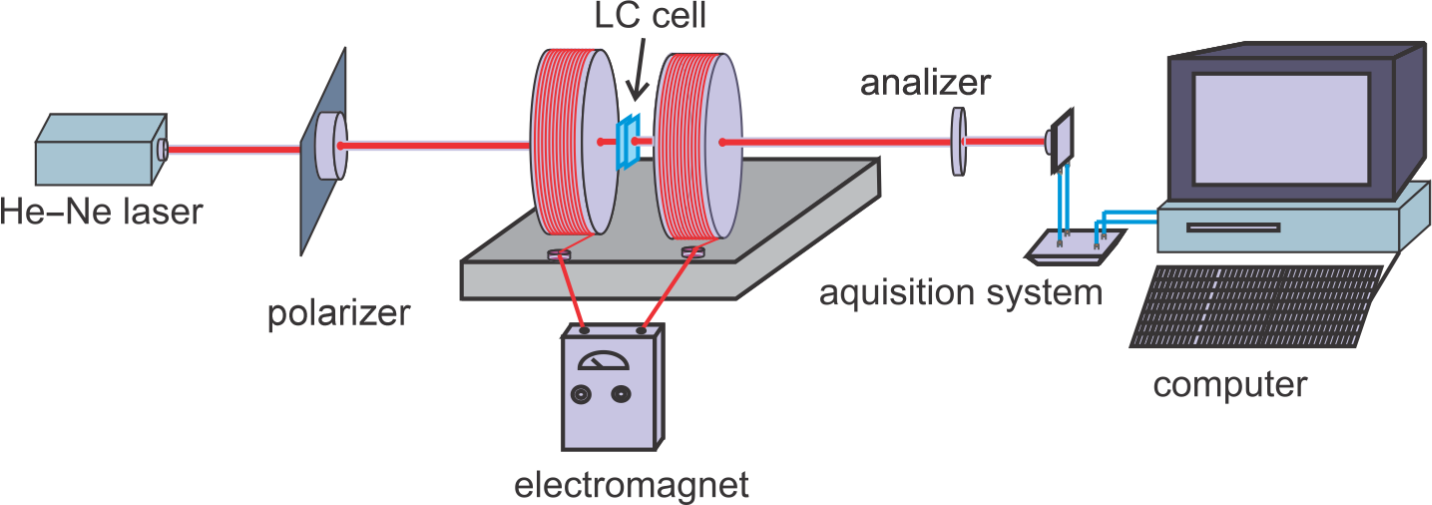
Experimental setup for the study of the internal organization of the mixture of 5CB with CoFe_2_O_4_.

**Figure 4:**
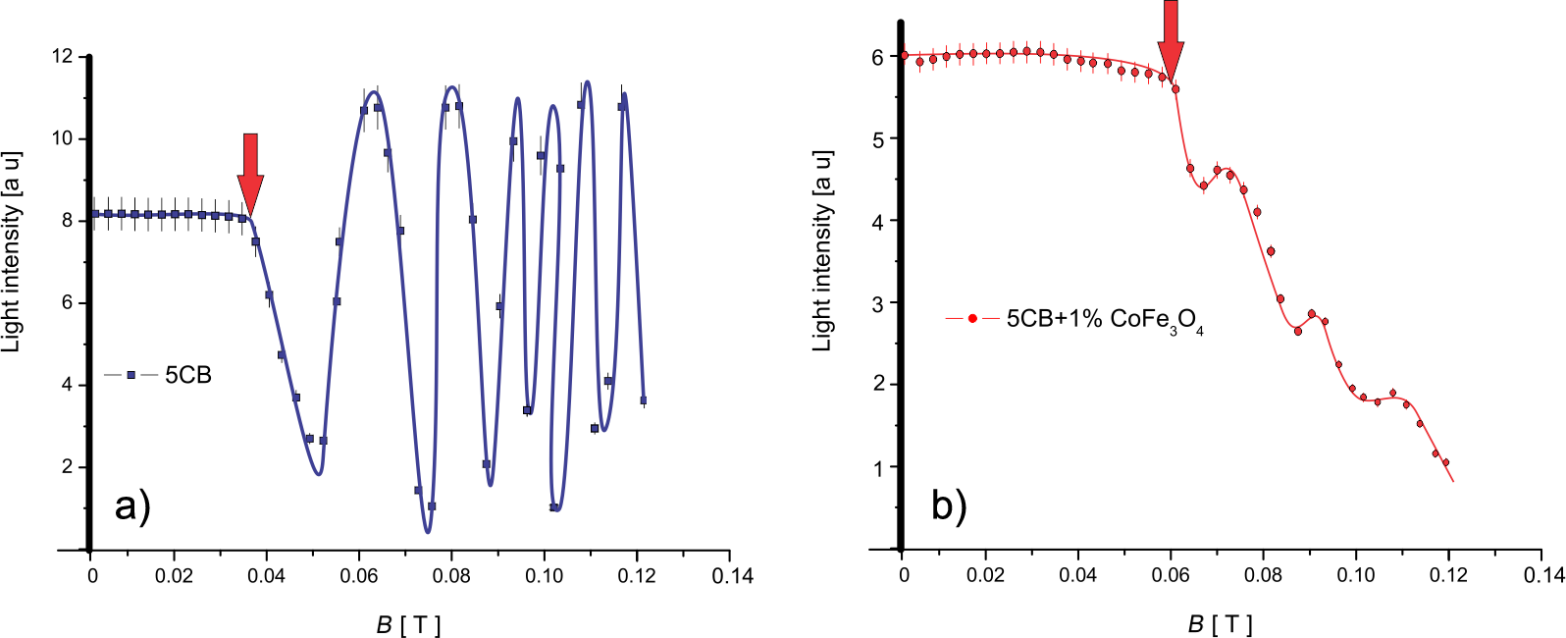
Magnetic Fréedericksz threshold for a) pure 5CB liquid crystals and b) the mixture of 5CB and CoFe_2_O_4_.

The experimental setup used for the measurement of the dynamic behavior of the nematic liquid crystal mixture is presented in Figure 3. An electromagnet with a maximum field strength of 1 T is used. Two crossed polarizers are placed outside the electromagnet and a 632.8 nm He–Ne laser is used for sample analysis. The emergent laser beam is recorded by a ThorLab photovoltaic cell and the data are collected to get real-time plots. The Fréedericksz transition threshold was determined by slowly increasing the applied field. It was found to be 0.047 T for the pure liquid crystal and 0.062 T for the cell containing the ferroparticles. The transition occurs when a significant change of the light intensity is measured while the strength of the applied field is increased (Figure 4).

**Figure 5:**
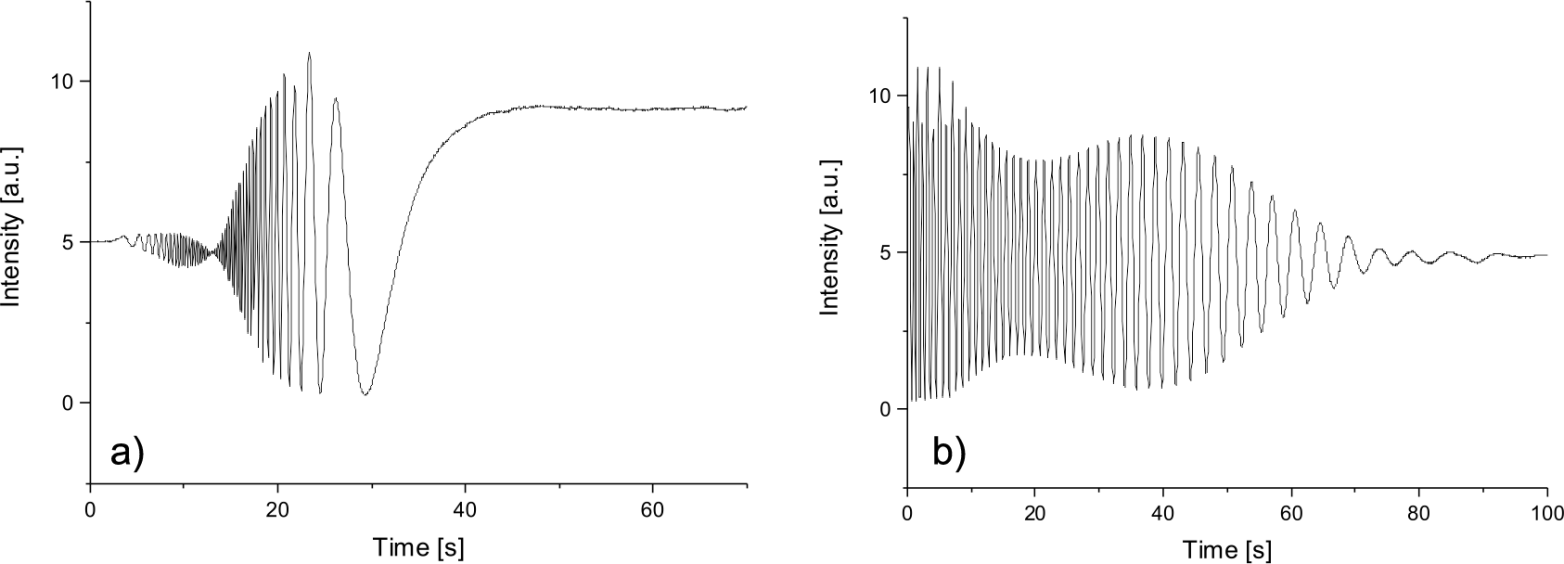
Experimental results plots for 0.1050 T magnetic field applied to the 5CB cell a) the field was applied, b) the field was disconnected.

In dynamic measurements a magnetic field higher than the critical Fréedericksz threshold was suddenly applied and the light intensity was recorded as a function of the time. The same measurement was carried out after the field was switched off. For a pure LC cell, a large number of local intensity maxima and minima was obtained (Figure 5).

**Figure 6:**
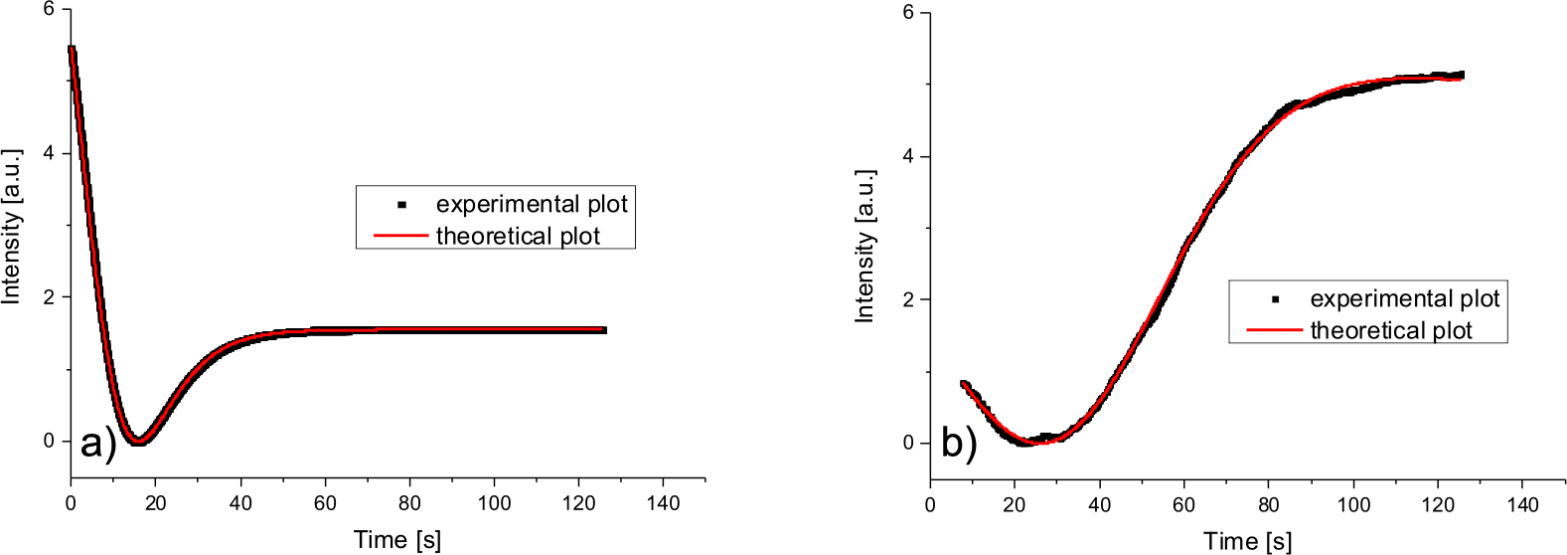
Experimental results and theoretical curves for a magnetic field of 0.1050 T applied to the mixture of 5CB and CoFe_2_O_4_ cell. a) The field was applied, b) the field was switched off.

When the mixture of 5CB and CoFe_2_O_4_ nanoparticles is used, just one intensity minimum is observed. A theoretical fit of these data that was made with Equation 7 and with θ given by Equation 9 and Equation 10 is showing good agreement with the experimental results (Figure 6).

Calculations performed for Equation 7 with the time dependence from Equation 9 indicates that the first minimum is obtained for an average deviation angle θ_m_(∞) = 0.1 rad when the field is switched on. As it can be observed from Figure 6, the experimental plot has just one minimum. Thus, no deviation angles larger than 0.1 rad can be obtained.

**Figure 7:**
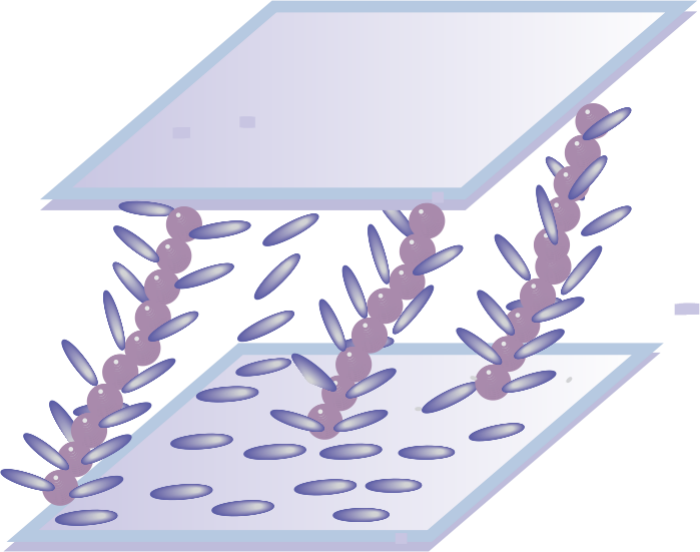
Fir branch-like structure in the cell with the mixture of 5CB with CoFe_2_O_4_.

Optical observations with a polarised microscope performed in a previous work [27] showed the presence of long nanoparticle chains inside the cell. Under the action of magnetic fields, these chains remain obliquely aligned between the support plates. The nematic molecules attached on these chains by strong anchoring forces can not change their orientation under the action of the magnetic field as they do in pure liquid crystal. Fir branch-like structures are obtained and the nematic molecular movement is limited (Figure 7).

We also observed a stable evolution for low magnetic fields (no secondary maxima and minima are obtained in low magnetic fields) and a quick response to field action. The relaxation times for this mixture were calculated using experimental results and the theoretical model described above. They are presented in Table 1 where τ_on_ is the relaxation time after the field is switched on, and τ_off_ is the relaxation time after the field is switched off.

**Table 1:** Relaxation times for the cell with the mixture of 5CB with CoFe_2_O_4_.

*B* (T)	τ_on_ (s)	τ_off_ (s)

0.1050 ± 0.0053	7.56 ± 0.01	32.39 ± 0.09
0.1189 ± 0.0059	4.27 ± 0.01	26.31 ± 0.13
0.1336 ± 0.0066	3.92 ± 0.02	20.76 ± 0.23

In pure nematic substances, the relaxation time τ_off_ does not depend on the applied field as long as it is higher than the Fréedericksz transition threshold. As it can be observed from Table 1, when ferro-particles are added, the relaxation time decreases with increase of the applied field.

## Conclusion

A new alignment was obtained in liquid crystal cells with added ferromagnetic nanoparticles as a consequence of strong interactions between ferromagnetic nanoparticles, nematic molecules and the support area. Using low concentrations of magnetic ferroparticles does not considerably affect the light transmission through the sample and improves the stability, proving to provide important benefits for magneto-optic devices. We obtained a stable structure with controllable phase modulation without backflow effect, and fast response. An important step was made to reduce the relaxation time, a major problem in magnetic devices well known for their slowness. The theoretical model applied is in good agreement with experimental data and can be used to design magneto-optic devices consisting of LCs and magnetic nanoparticles .
